# Supplementary material for: The causal role of circulating inflammatory markers in osteoporosis: a bidirectional Mendelian randomized study
Source: Front Immunol. 2024 Jul 18;15:1412298. doi: 10.3389/fimmu.2024.1412298 (PMC11291241; doi:10.3389/fimmu.2024.1412298)
Supplement: Supplementary file 1 [file DataSheet_1.zip › supplementary material/CIM-OSTEOPOROSIS /GCST90274803.csv_leave_one_out.pdf]

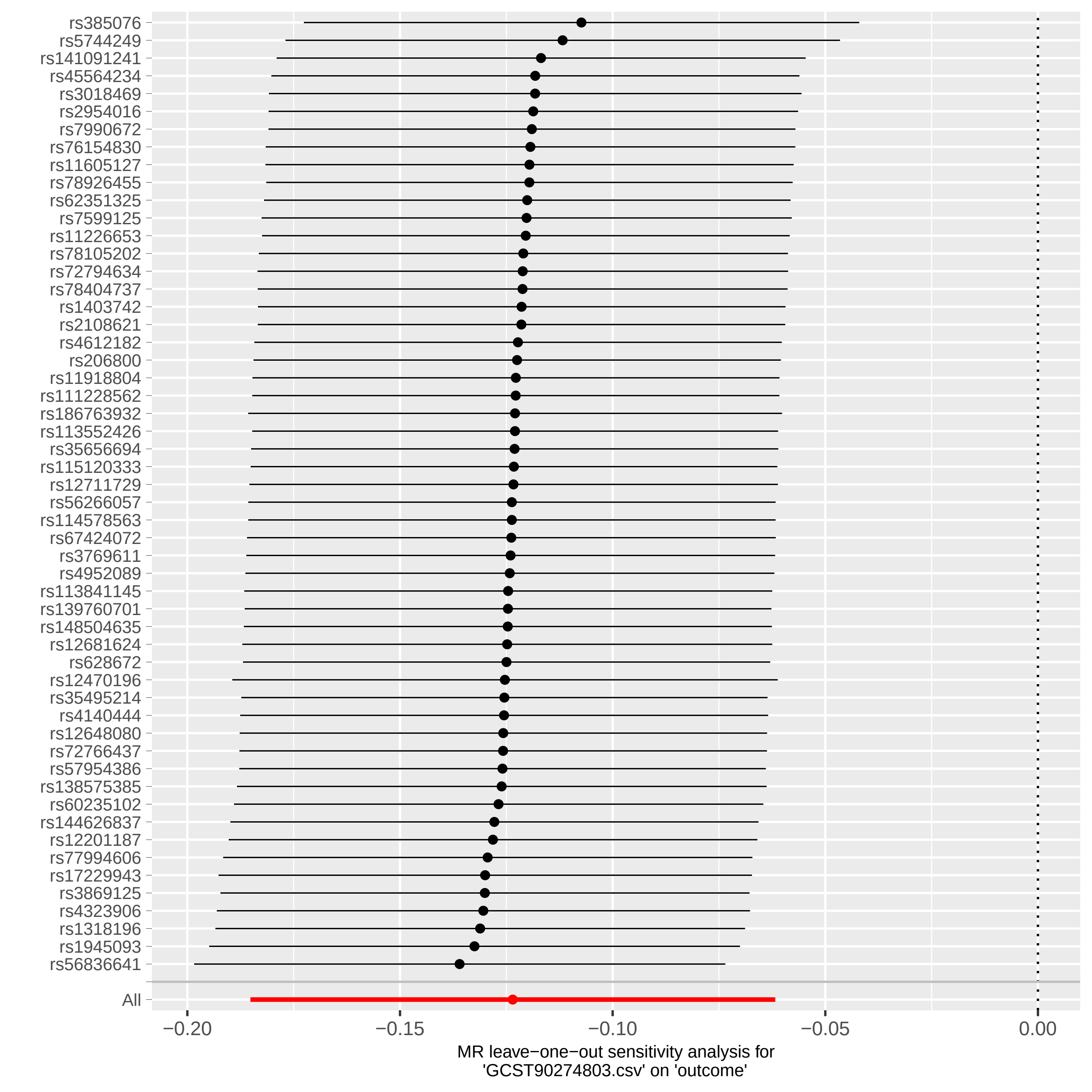
rs385076
rs5744249
rs141091241
rs45564234
rs3018469
rs2954016
rs7990672
rs76154830
rs11605127
rs78926455
rs62351325
rs7599125
rs11226653
rs78105202
rs72794634
rs78404737
rs1403742
rs2108621
rs4612182
rs206800
rs11918804
rs111228562
rs186763932
rs113552426
rs35656694
rs115120333
rs12711729
rs56266057
rs114578563
rs67424072
rs3769611
rs4952089
rs113841145
rs139760701
rs148504635
rs12681624
rs628672
rs12470196
rs35495214
rs4140444
rs12648080
rs72766437
rs57954386
rs138575385
rs60235102
rs144626837
rs12201187
rs77994606
rs17229943
rs3869125
rs4323906
rs1318196
rs1945093
rs56836641
All
−0.20
−0.15
−0.10
−0.05
0.00
MR leave−one−out sensitivity analysis for
'GCST90274803.csv' on 'outcome'
